# Unpacking the Influence of Abuse and Depression on Grades among Urban Ethnic Minority Adolescents and Young Adults

**DOI:** 10.5334/aogh.2478

**Published:** 2019-04-09

**Authors:** Perry Nagin, Anthony Salandy, Angela Diaz

**Affiliations:** 1 Boston Medical Center, US; 2 Mount Sinai Adolescent Health Center, Icahn School of Medicine at Mount Sinai, US

## Abstract

**Background:**

Both depression and a history of abuse have known negative consequences on the overall health of adolescents and young adults (AYAs). Research is not clear, however, on the interactive influence of abuse and depression on academic achievement, especially among AYAs of color.

**Objective(s):**

This study aims to assess the interactive influence of abuse and depression on academic grades among African American and Latino AYAs.

**Methods:**

The study sample was made up of 476 predominantly urban African American and Latino youth ages 14 to 24. Study participants completed a demographic questionnaire (which included self-reported grades) and the Beck Depression Inventory for Primary Care-Fast Screen. Screenings for abuse were done through three structured methods using the Childhood Maltreatment Interview Schedule-Short Form, a short-structured questionnaire, and a face-to-face unstructured interview with a clinical provider.

**Findings:**

Depression had a significant main effect on grades, while abuse did not. Abuse and depression had a significant interactive effect on grades in that non-depressed adolescents who reported abuse had an almost four point higher average grade score than their non-depressed counterparts who did not report abuse.

**Conclusions:**

Our findings highlight an unexpected effect in AYAs of color with a history of abuse but no history of depression, suggesting that perhaps there is something intrinsic to this group’s resilience or their support systems that protects both against depression and supports their academic achievement. In conclusion, abuse alone does not serve as a predictor of grade achievement. Further work should be done to determine influential factors behind this relationship, with recommendations for school-based counselors and medical providers to screen for depression along with abuse in AYAs in order to determine how best to support this population.

## Introduction

The adverse effects of childhood maltreatment on health outcomes and cognitive development have been studied; however, the impact on educational outcomes remain largely unexplored [1,2,3]. Education has often been linked to increased resilience, improved quality of health, and less risky decision making, and studies suggest that it appears to serve both a protective role against abuse [4] as well as a mitigating role against some of the negative effects once childhood abuse has occurred [5,6,7,8,9]. While this relationship has the potential to positively shape both interventions and targeted support for children suffering from abuse [10], studies also suggest that childhood maltreatment and adversity simultaneously act as barriers to remaining in school or achieving higher levels of education [3,11,12,13].

Current research supports that children who have experienced abuse are significantly more likely to suffer from depression, anxiety, substance abuse, and to demonstrate increased risk-taking behaviors [1,2,14,15,16,17,18,19,20,21,22,23,24,25]. These widespread effects often persist throughout adolescence and adulthood [25,26,27,28,29], and have the potential to largely impact a child’s educational achievement and ability to partake in and attend school [30,31,32].

Therefore, it is often difficult to parse out the association between depression and a history of abuse on the same outcome—educational achievement. While the effects of depression on academic achievement have been studied [3,11,12,13], the compounding effect of a history of childhood abuse on educational outcomes has largely not been explored in the United States, or in youth of color. Our objective, is to examine the role that a history of childhood abuse plays on academic achievement in AYAs of color in order to further unpack the association between childhood abuse, depression, and academic outcomes and to provide recommendations on how best to identify and address the needs of an at-risk population.

## Research Design and Methods

### Study Population

Our sample consists of adolescents and young adults who were recruited from a population of youth seeking general health services at the Mount Sinai Adolescent Health Center (MSAHC), a primary care clinic designed specifically for the needs of adolescents and young adults ages 10–24 years old. MSAHC provides free, confidential, and comprehensive integrated physical, sexual, reproductive, and behavioral health, as well as dental and optical services.

### Study recruitment

Participants were approached by a research assistant (RA) while waiting to see a medical provider. Participants were recruited with the following inclusion criteria: age of 12–24 years old and able to speak and read English. Institutional Review Board (IRB) approval was obtained from the Icahn School of Medicine at Mount Sinai prior to the data collection. Adolescents received two movie tickets upon the completion of their involvement in exchange for their time and participation.

### Sample Population

The sample initially included 532 participants who had completed a screening for childhood abuse history. Twenty-six study participants were missing relevant data and were excluded. An additional 30 participants were excluded because they had dropped out of school over 12 months, resulting in a final sample of 476. Participants included in the analytic sample and those excluded because of missing data were similar in all demographic characteristics, covariates, and prevalence of depression (p-values > 0.05). Demographics of our population are provided in Table 1.

**Table 1:** Baseline characteristics of adolescents who reported having experienced abuse and adolescents who reported not having experienced abuse.

Characteristics	Abuse	No Abuse
	%(N)	%(N)

**Age**		
≤14	4(19)	5(24)
15–17	22.5(107)	18.5(88)
18≥	27.5(131)	22.5(107)
**Gender**		
Female	46.4(221)	38.2(182)
Male	7.4(35)	7.1(34)
Transgender	0.2(1)	0.6(3)
**Race**		
Hispanic/Latin	29.4(140)	23.8(113)
Black	20.8(99)	19.7(94)
Asian/White	3.8(18)	2.5(12)
**Nativity Status**		
US Born	43.1(205)	39.3(197)
Non-US born	10.9(52)	6.7(32)
**Last grade completed**		
≤8th grade	4.6(22)	5.9(28)
9th grade	7.6(36)	5.9(28)
10th grade	6.1(29)	6.1(29)
11th grade	9.9(47)	8.0(38)
12th grade	9.5(45)	8.8(42)
College	16.4(78)	11.3(54)

### Safety Protocol

Each potential participant was informed during the consent process that any childhood abuse or suicidality disclosed during the research study would be shared with the participant’s medical provider before the participant left the clinic. Participants younger than 18 years of age were informed that if they disclosed childhood abuse, the MSAHC staff members would assess the history in detail and determine the necessary next steps regarding the need to report to Child Protection Services as mandated by law.

### Study Methods

Once participants consented to being in the study, they were first screened for a history of childhood abuse and, during the same interview, later completed a demographic questionnaire and the Beck Depression Inventory for Primary Care-Fast Screen (BDI-FS) [33], all prior to being seen by a medical provider. All participants completed the demographic questionnaire and the BDI-FS prior to receiving clinical services, which were then provided by their usual health care provider once the instruments were completed.

### Covariates

*Abuse*: The first covariate was self-reported retrospective history of childhood physical and sexual abuse that occurred before 17 years of age and was disclosed during the face-to-face unstructured interview or of any of the three structured screening methods of the Childhood Maltreatment Interview Schedule-Short Form (CMIS-SF) [34]. The CMIS-SF has been shown to have good internal consistency in adolescent and young adult populations [34]. Questions about parental substance abuse, domestic violence between parents, and emotional neglect of the participants were omitted to shorten the instrument, as these areas were not a focus of study. In the three structured interviews, information about *childhood physical abuse* was collected through the following questions: “Before you were 17 years of age: did a parent or guardian ever do something to you on purpose (for example, hit or punch or cut you, or push you down) that made you bleed or gave you bruises or scratches, or that broke bones or teeth? Did either of your parents or guardians get so mad at you that they hurt you physically? Did either of your parents or guardians use physical punishment for discipline?” Each question had answer choices of “Yes” or “No.” A participant was considered to have a history of physical abuse if they answered “Yes” to any of these questions.

For the face-to-face unstructured interview method, physical abuse information was assessed by asking participants: “How do your parents discipline you? How do they punish you? Do they ever physically hit you?” A positive determination of physical abuse was made if the participant described having been hit, punched, kicked, otherwise struck or pushed down, cut, bruised, made to bleed, scratched, having broken bones, broken teeth, or having been hurt physically. The unstructured interview covered the same issues as the three structured screens but incorporated the possibility of probing and asking other questions as needed.

For analytical purposes for this paper, participants were classified into three groups: (1) no abuse history (46% n = 232); (2) history of physical abuse only (29.6% n = 150); and (3) history of sexual abuse with or without physical abuse (25% n = 124). Hereafter, physical and sexual abuse are classified together as history of abuse (yes or no).

*Depression*: The BDI-FS [33] is a validated 7-item self-report instrument with good psychometric properties developed to assess depression within a medical population. The symptoms measured include: sadness, pessimism, past failure, loss of pleasure, self-dislike, self-criticalness, and suicidal thoughts [35]. Each item is marked 0 through 3 using a Likert-like scale, and 21 is the highest score if the study participant responded with a 3 to all of the items. Zero is the lowest score if the patient answered each question with a 0. The cut points were as follows: 0–3 is minimal; 4–8 is mild; 9–12 is moderate, and 13–21 is severe depression [36]. Scores with ≥4 were recoded as “1”—experiencing depression, and scores of <4 were recoded as “0”—not experiencing depression. The BDI-FS was also used to screen for suicidality.

### Outcome measure

Participants were asked: “What was the last grade that you completed in school?” and given the choices of 4th, 5th, 6th, 7th, 8th, 9th, 10th, 11th, 12th grades and college. The last grade completed was categorized into eighth grade or lower, ninth, tenth, eleventh, twelfth, and college. Study participants’ school performance in the past year was assessed by asking: “In the past year, what best represents your average grade?” Choices given and included in the analysis were: 90–100%; 80–89%; 70–79%; 65–69%; or 64% or lower and was recorded as 4, 3, 2, 1, and 0, respectively. Students who self-reported dropping out of school and not graduating were excluded from the study (n = 30), leaving a final sample of 476.

## Results

Overall, findings indicated no significant difference between having experienced abuse and not having experienced abuse across all demographic factors. However, nativity status did approach significance (p = .06). Specifically, youth who were born outside of the US reported experiencing abuse far greater than their US born counterparts (62% non-US born compared to 52% US born).

To examine the influence of abuse and depression on past year’s grades, a two-way ANOVA was conducted. Residual analysis was performed to test for the assumptions of the two-way ANOVA. Outliers were assessed by inspection of a boxplot, normality was assessed using Shapiro-Wilk’s normality test for each cell of the design, and homogeneity of variances was assessed by Levene’s test. There were no outliers, residuals were normally distributed (p > .05), and there was homogeneity of variances (p = .96). There was a statistically significant main effect for depression, F(1, 472) = 15.97, p < .01, partial η^2^ = .03. There was a trending main effect for abuse, F(1, 472) = 3.785, p = .05, however, it was not significant below the .05 level, partial η^2^ = .008.

There was a statistically significant interaction between abuse and depression on grades, F(1, 472) = 4.26, p < .05, partial η^2^ = .009 (see Table 2). An interaction contrast was run that compared the difference in mean grades for depressed and abused youth, depressed and non-abused youth, non-depressed and abused, and non-depressed and non-abused youth. Pairwise comparisons were run for each simple main effect with reported 95% confidence intervals and p-values Bonferroni-adjusted within each simple main effect.

**Table 2:** Two-way ANOVA considering the effects of abuse, depression, and the interaction term on grades.

Source	Sum of Squares	df	Mean Square	F	p-value	Partial Eta Squared

Corrected Model	15.610^a^	3	5.203	6.638	.000	.040
Intercept	2478.845	1	2478.845	3162.456	.000	.870
abuse	2.967	1	2.967	3.785	.052	.008
depressed	12.442	1	12.442	15.874	.000	.033
abuse*depressed	3.340	1	3.340	4.262	.040	.009
Error	369.970	472	.784			
Corrected Total	385.580	475				

^a^ R Squared = .040 (Adjusted R Squared = .034).

**Figure 1:**
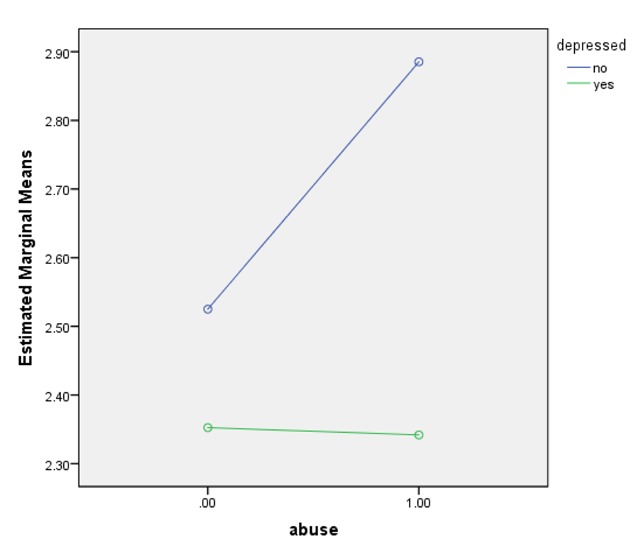
Estimated means by the model with the interaction term (abuse and depression on grades).

Mean and (standard errors) are reported. Mean grade scores for depressed adolescents who reported experiencing abuse and not experiencing abuse were 2.34 (.06) and 2.35 (.08), respectively, whereas, mean grade scores of non-depressed adolescents who reported experiencing abuse and not experiencing abuse were 2.88(.11) and 2.53(.10), respectively (see Table 3). Non-depressed adolescents who reported no abuse had 36 (95% CL, –.53 to –.18) lower grades scores than their non-depressed counterparts who reported abuse—a statistically significant difference, p < .01 (see Figure 1).

**Table 3:** Comparative means of grades under the two-way ANOVAL model with the interaction model.

Abuse	Depressed	Mean	Std. Error	95% Confidence Interval	
				Lower Bound	Upper Bound

.00	no	2.525^b^	.099	2.330	2.720
	yes	2.353^b^	.075	2.205	2.500
1.00	no	2.885^a^	.113	2.662	3.108
	yes	2.342^b^	.063	2.218	2.466

* Different alphabets mean significantly different values at a type one error rate of 0.05. P-value (model) < .01.

## Conclusion

Our results suggest that a history of abuse alone did not serve as a significant predictor of educational outcomes as measured through grade points achieved; depression alone, in comparison, did indeed demonstrate a significant impact on grades. Youth with a history of abuse and no evidence of depression, however, reported a significantly higher average grade achieved than those with neither a history of abuse nor depression.

It is unclear at this point as to what may be driving this finding, however; perhaps there are underlying factors shared regarding resilience in a group of AYAs of color who experienced abuse and yet are not depressed. Studies have demonstrated that positive intervention and support systems can serve to build resilience in youth suffering from a variety of adverse events, and to mitigate known negative consequences for children [37,38]. Perhaps their lack of depression is a reflection of the support systems in their lives, such as an academic community that serves as an outlet, or intrinsic qualities that have assisted in their abilities to build resilience. In contrast, perhaps these results are a reflection of the variation in measures of abuse, what types of abuse may correlate further with depression, or variation in what is considered abuse by youth in this population. Screening methods attempted to differentiate between corporal punishment and abuse based on levels of physical injury; however, perhaps these findings reflect differences in perceptions of abuse. Additionally, the difference between AYAs of color born in the United States and outside the United States reporting abuse suggests that perhaps there are further cultural differences in definitions of abuse, and therefore the consequences, in this population.

Limitations of our study include errors associated with such reliability of self-reported histories of abuse, as well as the varieties in classification of behavior as abuse [39]. Additionally, in this setting, we chose to collapse sexual and physical abuse for statistical purposes, which are often studied separately; neglect was also not studied. We recognize the variances in the impact of those types of abuse, and further work should be done to study those differences more closely. Additionally, we chose to exclude those who had dropped out in order to compare academic achievement by grades obtained. This likely excluded a vulnerable population; however, we were unable to obtain further information as to the reason for dropping out. An additional limitation is self-reported grades, as well as basing academic achievement on only one outcome. Further studies should be done to address a broader definition of academic outcomes in this setting.

In conclusion, abuse alone does not serve as a predictor of a minority youth’s academic achievement, and further research is needed to understand how certain youth remain not only resilient to depression but able to excel in comparison to peers without a history of abuse. Educators and health care providers should continue the ongoing supports for those suffering from both depression and abuse, with a sensitive eye for screening and intervention for students struggling academically. Lastly, further studies should address the factors that play a role in increasing the resilience of an otherwise at-risk population in hopes of providing better support systems for future youth.

## References

[1] Brown J, Cohen P, Johnson JG and Smalles EM. Childhood abuse and neglect: Specificity of effects of adolescent and young adult depression and suicidality. 1999 12; 38(12): 1490–6. DOI: 10.1097/00004583-199912000-00009 10596248

[2] Widom C, DuMont K and Czaja SJ. A prospective investigation of major depressive disorder and domorbidity in abused and neglected children grown up. Arch Gen Psychiatry. 2007; 64(1): 49–56. DOI: 10.1001/archpsyc.64.1.49 17199054

[3] Flores G and Lesley B. Children and US federal policy on health and health care: Seen but not heard. 2014 12; 168(12): 1155–63. DOI: 10.1001/jamapediatrics.2014.1701 25329439

[4] Mersky JP, Berger LM, Reynolds AJ, et al. Risk factors for child and adolescent maltreatment: A longitudinal investigation of a cohort of inner-city youth. 2009 2; 14(1): 73–88. DOI: 10.1177/1077559508318399 PMC4545632 18596199

[5] Romans SE, Martin JL, Anderson JC, et al. Factors that mediate between child sexual abuse and adult psychological outcome. Psychol Med. 1995; 25(1): 127–42. DOI: 10.1017/S0033291700028154 7792348

[6] Edmond T, Auslander W, Elza D, et al. Signs of resilience in sexually abused girls in the foster care system. J of Child Sexual Abuse. 2006; 15(1): 1–28. DOI: 10.1300/J070v15n01_01 16551583

[7] Williams J and Nelson-Gardell D. Predicting resilience in sexually abused adolescents. Child Abuse & Neglect. 2012; 36(1): 53–63. DOI: 10.1016/j.chiabu.2011.07.004 22265933

[8] Bauldry S, Shanahan MK, et al. A life course model of self-rated health through adolescence and young adulthood. Soc Sci Med. 2012; 75(7): 1311–1320. DOI: 10.1016/j.socscimed.2012.05.017 22726620 PMC4297471

[9] As-Sanie S, Clevenger LA, Geisser ME, et al. History of abuse and its relationship to pain experience and depression in women with chronic pelvic pain. Am J Obstet Gynecol. 2014; 210(4): 317. e1–317.e8 DOI: 10.1016/j.ajog.2013.12.048 24412745 PMC4086742

[10] LeBlanc JB, Brabant S and Forsyth CJ. The meaning of college for survivors of sexual abuse: Higher education and the older female college student. Am J Orthopsychiatry. 1996; 66(3): 468–73. DOI: 10.1037/h0080197 8827270

[11] Schilling EA, Aseltine RH and Gore S. Young women’s social and occupational development and mental health in the aftermath of child sexual abuse. Am J Community Psychol. 2007; 40: 109–124. DOI: 10.1007/s10464-007-9130-3 17557204

[12] Lansford JE, Dodge KA, Pettit GS, et al. A 12-year prospective study of the long-term effects on early child physical maltreatment on psychological, behavioral, and academic problems in adolescence. Arch Pediatr Adolesc Med. 2002; 156(8): 824–830. DOI: 10.1001/archpedi.156.8.824 12144375 PMC2756659

[13] Currie J and Widom CS. Long-term consequences of child abuse and neglect on adult economic well-being. Child Maltreat. 2010 5; 15(2): 111–120. DOI: 10.1177/1077559509355316 20425881 PMC3571659

[14] Briere J. Prevalence and psychological sequelae of self-reported childhood physical and sexual abuse in a general population sample of men and women. Child Abuse and Negl. 2003; 27(10): 1205–22. DOI: 10.1016/j.chiabu.2003.09.008 14602100

[15] Cannon EA, Bonomi AE, Anderson ML, et al. Adult health and relationship outcomes among women with abuse experiences during childhood. Violence Vict. 2010; 25(3): 291–305. DOI: 10.1891/0886-6708.25.3.291 20565002

[16] Lown EA, Nayak MB, Korcha RA, et al. Child physical and sexual abuse: A comprehensive look at alcohol consumption patterns, consequences, and dependence from the National Alcohol Survey. Alcohol Clin Exp Res. 2011 2; 35(2): 317–325. DOI: 10.1111/j.1530-0277.2010.01347.x 21083668 PMC3026876

[17] Felittie VJ, et al. Relationship of childhood abuse and household dysfunction to many of the leading causes of death in adults: The Adverse Childhood Experiences (ACE) Study. American Journal of Preventive Medicine. 1998; 14(4): 245–258. DOI: 10.1016/S0749-3797(98)00017-8 9635069

[18] Hahm HC, Kolaczyk E, Lee Y, et al. Do Asian-American women who were maltreated as children have a higher likelihood for HIV risk behaviors and adverse mental health outcomes? 2012 1; 22(1): e35–e43. DOI: 10.1016/j.whi.2011.07.003 PMC3236805 21872488

[19] Norman RE, Byambaa M, De R, et al. The long-term health consequences of child physical abuse, emotional abuse, and neglect: A systematic review and meta-analysis. PLoS Med. 2012 11; 9(11): e1001349 DOI: 10.1371/journal.pmed.1001349 23209385 PMC3507962

[20] Bruce LC, Heimberg RG, Blanco C, et al. Childhood maltreatment and social anxiety disorder: Implications for symptoms severity and response to pharmacotherapy. Depress Anxiety. 2012 2; 29(2): 132–139. DOI: 10.1002/da.20909 PMC3314083 22065560

[21] Shin SH and Miller DP. A longitudinal examination of childhood maltreatment and adolescent obesity: Results from the National Longitudinal Study of Adolescent Health (AddHealth) Study. 2012; 36(2): 84–94. DOI: 10.1016/j.chiabu.2011.08.007 22398304

[22] Reiser SJ, McMillan KA, Wright KD, et al. Adverse childhood experiences and health anxiety in adulthood. Child Abuse & Neglect. 2014; 38(3): 407–413. DOI: 10.1016/j.chiabu.2013.08.007 24011493

[23] Martins CM, Von Werne Baes C, Tofoli SM, et al. Emotional abuse in childhood is a differential factor for the development of depression in adults. 2014 11; 202(11): 774–82. DOI: 10.1097/NMD.0000000000000202 25268154

[24] Diaz A, Simantov E and Rickert VI. Effects of abuse on health: Results of national survey. Arch Pediatr Adolesc Med. 2002; 156(8): 811–7. DOI: 10.1001/archpedi.156.8.811 12144373

[25] Diaz A and Petersen AC. Institute of Medicine Report: New directions in child abuse and neglect research. JAMA Pediatrics. 2014; 168(2): 101–2. DOI: 10.1001/jamapediatrics.2013.4560 24217391

[26] Johnson JG, et al. Childhood verbal abuse and risk for personality disorders during adolescence and early adulthood. 2001 1; 42(1): 16–23. DOI: 10.1053/comp.2001.19755 11154711

[27] Cecil CA, Viding E, Barker E, et al. Double disadvantage: The influence of childhood maltreatment and community violence exposure on adolescent mental health. J Child Psychol Psychiatry. 2014; 55(7): 839–48. DOI: 10.1111/jcpp.12213 24611776

[28] Calvete E. Emotional abuse as a predictor of early maladaptive schemas in adolescents: Contributions to the development of depressive and social anxiety symptoms. 2014; 38(4): 735–746. DOI: 10.1016/j.chiabu.2013.10.014 24252743

[29] Kiser LJ, Smith C, et al. Effects of the child-perpetrator relationship on mental health outcomes of child abuse: It’s (not) all relative. Child Abuse & Neglect. 2014; 38: 1083–1093. DOI: 10.1016/j.chiabu.2014.02.017 24661693

[30] de Ridder DT, Lensvelt-Mulders G, Finkenauer C, et al. Taking stock of self-control: A meta-analysis of how trait self-control relates to a wide range of behaviors. Pers Soc Pyschol Rev. 2012; 16(1): 76–99. DOI: 10.1177/1088868311418749 21878607

[31] Basch CE. Healthier students are better learners: A missing link in school reforms to close the achievement gap. J Sch Health. 2001; 81(19): 593–8. 10.1111/j.1746-1561.2011.00632.x 21923870

[32] Freudenberg N and Ruglis J. Reframing school dropout as a public health issue. Prev Chronic Dis. 2007; 4(4). PMC2099272 17875251

[33] Beck AT, Steer RA and Brown GK. Beck Depression Inventory–Fast Screen for Medical Patients The Psychological Corporation; 2000 San Antonio, TX.

[34] Briere, J. Child Abuse Trauma: Theory and Treatment of the Lasting Effects. Newbury Park, CA: Sage; 1992.

[35] Strauss E, Sherman EMS and Spreen O. A Compendium of Neuropsychological Tests: Administration, Norms, and Commentary. 3rd. ed New York, NY: Oxford University Press; 2006.

[36] Whiston S and C Eder K. Review of the BDI-FastScreen for Medical Patients In: The seventeenth mental measurements yearbook, Plake BS, Impara JC and Spies RA (Eds.). Lincoln, NE: Buros Institute of Mental Measurements; 2003 http://web.ebscohost.com.ezp.waldenulibrary.org/.

[37] Widom C and Shepard R. Accuracy of adult recollections of childhood victimization: Part 1. Childhood physical abuse. Psychological Assessment. 1996; 8(4): 412–21. DOI: 10.1037/1040-3590.8.4.412

[38] Widom C and Morris S. Accuracy of adult recollections of childhood victimization: Part 2. Childhood sexual abuse. Psychological Assessment. 1997; 9(1): 34–46. DOI: 10.1037/1040-3590.9.1.34

[39] Institute of Medicine; National Research Council. New Directions in Child Abuse and Neglect Research. Washington, DC: The National Academies Press; 2013. 24757747

